# Metabolic Recycling Enhances Proliferation in MYC-Transformed Lymphoma B Cells

**DOI:** 10.1002/adbi.202200233

**Published:** 2022-11-23

**Authors:** Cissy Zhang, Giang Hoang, Nabeel Attarwala, Arthur J. L. Cooper, Ryoichi Asaka, Anne Le

**Affiliations:** Department of Pathology, Johns Hopkins University School of Medicine, Baltimore, MD 21205, USA; Department of Pathology, Johns Hopkins University School of Medicine, Baltimore, MD 21205, USA; Department of Pathology, Johns Hopkins University School of Medicine, Baltimore, MD 21205, USA; Department of Biochemistry and Molecular Biology, New York Medical College, Valhalla, NY 10595, USA; Department of Pathology, Johns Hopkins University School of Medicine, Baltimore, MD 21205, USA; Department of Pathology, Johns Hopkins University School of Medicine, Baltimore, MD 21205, USA; Department of Oncology, Johns Hopkins University School of Medicine, Baltimore, MD 21205, USA; Department of Chemical and Biomolecular Engineering, Johns Hopkins University Whiting School of Engineering, Baltimore, MD 21218, USA

**Keywords:** ^13^C_6_-glucose labeling, cancer metabolism, glucose metabolism, mass spectrometry, metabolic recycling, metabolomics, MYC-transformed lymphoma B cells

## Abstract

Relapses negatively impact cancer patient survival due to the tumorigenesis ability of surviving cancer cells post-therapy. Efforts are needed to better understand and combat this problem. This study hypothesized that dead cell debris post-radiation therapy creates an advantageous microenvironment rich in metabolic materials promoting the growth of remaining live cancer cells. In this study, live cancer cells are co-cultured with dead cancer cells eradicated by UV radiation to mimic a post-therapy environment. Isotopic labeling metabolomics is used to investigate the metabolic behavior of cancer cells grown in a post-radiation-therapy environment. It is found that post-UV-eradicated dead cancer cells serve as nutritional sources of “off-the-shelf” and precursor metabolites for surviving cancer cells. The surviving cancer cells then take up these metabolites, integrate and upregulate multiple vital metabolic processes, thereby significantly increasing growth in vitro and probably in vivo beyond their intrinsic fast-growing characteristics. Importantly, this active metabolite uptake behavior is only observed in oncogenic but not in non-oncogenic cells, presenting opportunities for therapeutic approaches to interrupt the active uptake process of oncogenic cells without affecting normal cells. The process by which living cancer cells re-use vital metabolites released by dead cancer cells post-therapy is coined in this study as “metabolic recycling” of oncogenic cells.

## Table of contents entry

This study finds that a post-ultraviolet-therapy environment containing dead cancer cells promotes the proliferation of live cancer cells by serving as a source of “off-the-shelf” and precursor metabolites which the live cancer cells can absorb and incorporate into their metabolism. This phenomenon is only observed in oncogenic cells, but not in non-oncogenic cells, providing a potential target for cancer therapy.

## Graphical Abstract

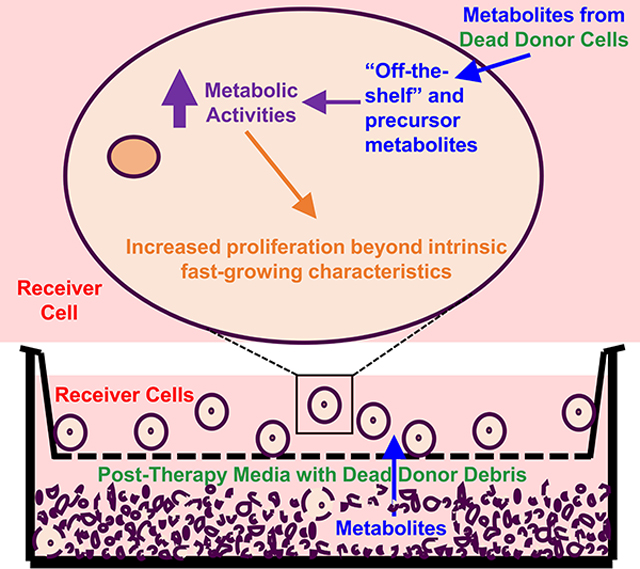

## 1. Introduction

The tumorigenesis ability of surviving cancer cells post-therapy leads to relapse and thus negatively impacts patient survival.^[1]^ After chemotherapy or radiotherapy, there is usually a low percentage of intact living cancer cells remaining at the tumor site surrounded by dead cancer cell debris. We hypothesize that this dead cell debris creates a microenvironment rich in metabolite and energy sources at the tumor site, promoting growth of the surviving cancer cells in the vicinity of the irradiated, dead cancer cells, thus helping to promote the growth of the remaining living cancer cells beyond their intrinsic fast-growing characteristics.

In this study, to mimic a post-therapy microenvironment, *MYC*-transformed human lymphoma B cells (or P493 *MYC*-ON cells containing a tetracycline-repressible *MYC* construct),^[2–7]^ were completely eradicated by UV radiation, and henceforth referred to as *dead donor cells*. Live, intact P493 cells (*receiver cells*), were then cultured in this newly created post-therapy environment. After first observing the growth-promoting effect of the post-therapy environment on the receiver cells both in vitro and in vivo, we used stable isotope-resolved metabolomics (SIRM) with labeled ^13^C_6_-glucose to investigate how this specific environment increased the proliferation of cancer cells beyond their intrinsic fast-growing characteristics. We discovered that post-therapy dead cancer cells serve as nutritional sources of “off-the-shelf” and precursor metabolites for the live cancer cells to take up and utilize and metabolize. This led to an overall increase in metabolite intensities and significantly increased the growth of cancer cells grown in a post-therapy medium compared to those grown in control medium (without dead donor cells). Finally, by performing the metabolomics experiments described above using non-oncogenic P493 *MYC*-OFF cells generated by suppressing *MYC* expression in *MYC*-ON cells using tetracycline,^[2–7]^ we confirmed that the active uptake of metabolites from the post-therapy medium is an oncogenic behavior. This work shows for the first time that metabolites derived from dead cancer cells may be utilized by live cancer cells to promote growth and proliferation.

## 2. Main Text

**Figure 1.**
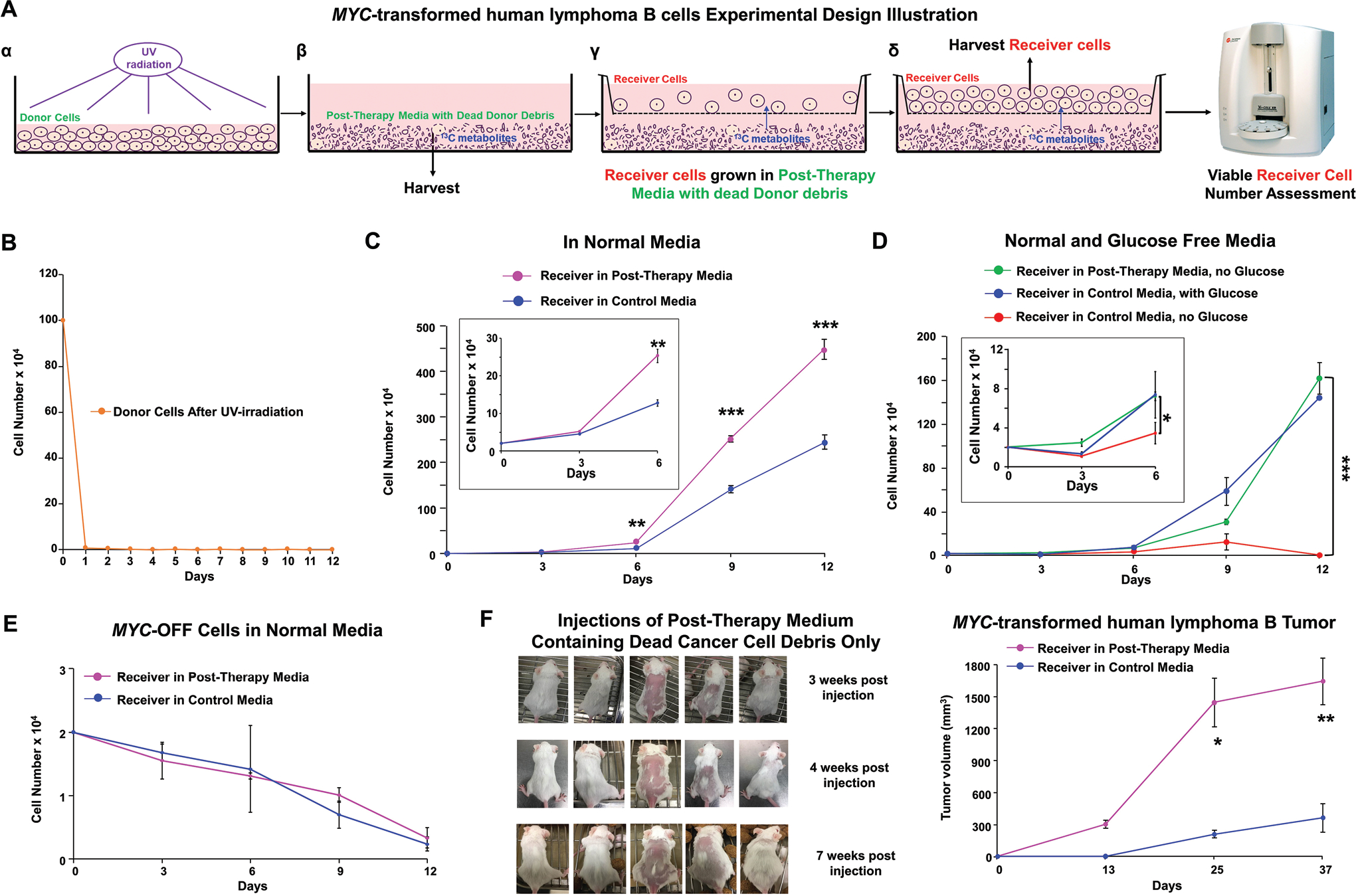
A) α) Donor cells were plated onto the bottom compartment of the transwell plate and subjected to UV radiation. β) Post-therapy medium with dead donor debris was harvested immediately, and at 24, 48, 72 h post-UV irradiation. γ) A membrane was inserted into each plate, onto which receiver cells were plated. δ) Proliferation and metabolism of receiver cells were assessed. B) Cell viability of donor cells post-UV irradiation. Cell numbers were assessed every day for 12 days. C) Receiver cell proliferation in normal post-therapy medium (pink line) compared to that in control medium (blue line) and D) receiver cell proliferation in glucose-free post-therapy medium (green line) compared to that in glucose-free control medium (red line) and that in control medium (blue line). Cell numbers were assessed every 3 days for 12 days. Zoomed-in graphs for the first 6 days are also presented due to a high number of cells at later dates. E) Effect of the post-therapy medium containing dead *MYC*-OFF cell debris on the growth of *MYC*-OFF receiver cells. Cell numbers were assessed every 3 days for 12 days. F) Effect of post-therapy medium containing dead cancer cell debris on the growth of receiver cells in vivo. Left: Mice with no tumor formation. Right: Receiver cells suspended in post-therapy medium (pink line) or in control medium after UV irradiation (blue line) were injected into the backs of SCID mice. Tumor sizes were measured 13, 25, and 37 days post-injection. Data are shown as mean ± SEM (standard error of mean) (*n* = 3–6 per group in vitro or *n* = 16–20 in vivo). *p < 0.05, **p < 0.01, ***p < 0.001 (Student’s *t-*test) where indicated. Experiments were replicated twice with similar results. Data from one set of experiments are shown.

We first sought to investigate whether the post-therapy environment could indeed promote the growth and proliferation of the live receiver cells. This was done by growing the receiver cells in a post-therapy medium containing debris of dead donor cells eradicated by UV radiation (Figure 1A-α,B) at a ratio of 1 live receiver cell to 50 dead donor cells, using a transwell with a permeable membrane between donor cell and receiver cell compartments. Although UV radiation is non-ionizing, it has been used in many previous studies to kill cells.^[8–12]^ The post-therapy medium containing dead donor cell debris was placed in the bottom compartment of the transwell, and live receiver cells in the top compartment, where their growth was evaluated every 3 days (Figure 1A-γ). The permeable separation membrane has a pore size diameter (0.4 μm) smaller than the diameter of lymphoma B cells (12–14 μm).^[13]^ As a result, the transwell prevents the exchange of whole cells, preventing surviving donor cells (if any) from interfering with the accurate assessment of receiver cell numbers. We observed a significant increase in the proliferation of receiver cells cultured in this post-UV-therapy medium containing dead donor cell debris as compared to receiver cells grown in UV-irradiated control medium lacking dead donor cell debris with no significant change in the average diameter of the cells, which were all ≈15 μm (Figure 1C).

Next, we evaluated the extent to which post-therapy medium containing dead donor cell debris can support live cancer cell growth by withdrawing one of the crucial fuel sources necessary for cancer cell proliferation, namely, glucose. Receiver cells were grown in a glucose-free post-therapy medium to determine whether such a medium could sustain cancer cell growth, even in the absence of glucose. While the glucose-free control medium compromised the growth of cancer cells (Figure 1D, red line vs blue line), the glucose-free *post-therapy* medium completely restored the growth of cancer cells, which reached a growth level comparable to those in the full-glucose control medium (Figure 1D, blue line vs green line). Taken together, these findings demonstrate that the post-therapy environment containing dead donor cell debris promoted the proliferation of living cancer cells in both standard and glucose-free medium. After confirming the growth-promoting effect of a post-therapy environment for oncogenic *MYC*-ON cells, we repeated the in vitro experiments with non-oncogenic *MYC*-OFF cells (P493 cells treated with 0.1 μg mL^−1^ tetracycline to suppress the *MYC* oncogene). We found no difference in receiver cells grown in the post-therapy medium as compared to the control medium (Figure 1E), the growth curve of which corroborates those from several previous studies for *MYC*-OFF cells.^[14–16]^ The lack of a difference in cell proliferation between the two groups demonstrates that, unlike the effect on oncogenic cells, the post-therapy environment does not have a growth-promoting effect on non-oncogenic *MYC*-OFF cells.

This newly discovered growth-promoting effect of the post-therapy environment in vitro prompted us to confirm the finding in vivo. While our previous study established that 20 million P493 lymphoma B cells were necessary to obtain optimal tumorigenesis in a xenograft model in 10–15 days,^[3]^ here we injected only 1 million receiver cells per tumor to investigate how effectively a post-therapy environment containing dead donor cell debris could promote tumorigenesis. Using the same ratio as we did in the in vitro experiments (1 live receiver to 50 donor cells), we first confirmed that mice injected with 50 million UV-irradiated donor cells (post-therapy medium) did not develop tumors over 7 weeks (Figure 1F, left). Simultaneously, we monitored this same batch of post-therapy medium to assess whether any live donor cells had survived UV radiation using a Vi-Cell XR Cell Viability Analyzer and found no viable cells. Next, we injected mice with 1 million live P493 receiver cells suspended in a post-therapy medium containing debris from 50 million dead donor cells. This resulted in both an early tumor development and a fivefold increase in tumor size compared to the injection of receiver cells suspended in the control medium (Figure 1F, right). This dramatic outcome strongly demonstrates the growth enhancement effect of the post-therapy environment containing dead donor cell debris on tumors.

**Figure 2.**
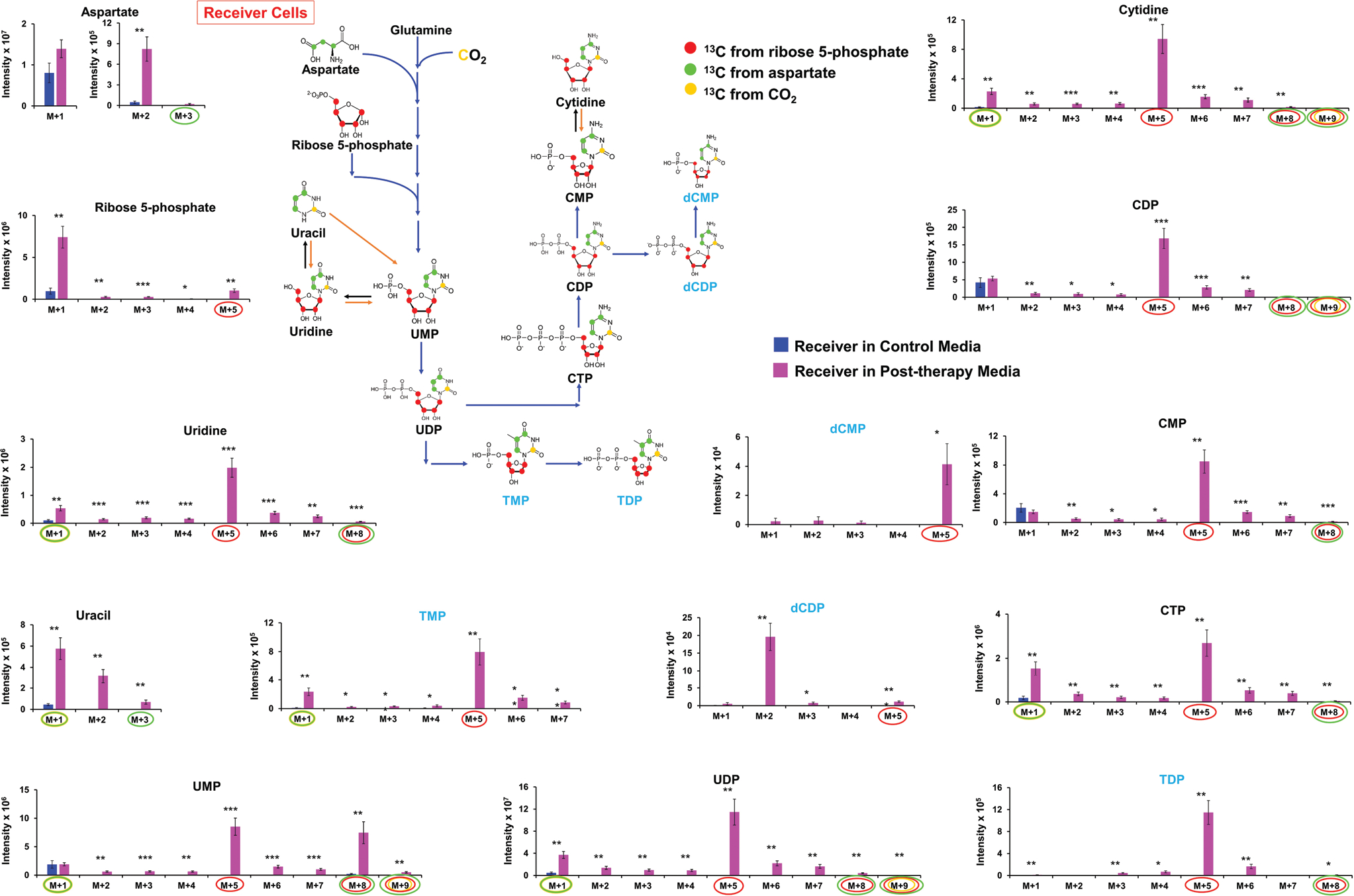
Isotopologues of ^13^C-labeled pyrimidine metabolites of receiver cells grown in post-therapy medium or in control medium and illustration of the corresponding pyrimidine metabolism pathways. Metabolite intensities of each isotopologue of receiver cells grown in post-therapy medium containing the dead donor cell debris (previously grown with ^13^C-labeled glucose) for 72 h are shown as pink bars; metabolite intensities of each isotopologue of receiver cells grown in control medium are shown as blue bars. Deoxyribonucleotide metabolites are shown in light blue. Other pyrimidine metabolites are shown in black. Red dots represent labeled-^13^C from ribose 5-phosphate. Green dots represent labeled-^13^C from aspartate. Yellow dots represent labeled-^13^C from CO_2_. Blue arrows indicate de novo synthesis, black arrows indicate catabolism, and orange arrows indicate salvage of the pyrimidine metabolites. The (m+5) isotopologues shown in red circles originate from (m+5) ribose 5-phosphate. The (m+3) isotopologues shown in green circles originate from (m+3) aspartate. The (m+1) isotopologues shown in green circles with yellow outlines originate from either (m+1) aspartate or (m+1) CO_2_. The (m+8) isotopologues shown in a combination of red and green circles originate from (m+3) aspartate combined with (m+5) ribose 5-phosphate. The (m+9) isotopologues shown in a combination of red, green, and yellow circles originate from (m+1) CO_2_ combined with (m+3) aspartate combined with (m+5) ribose 5-phosphate. Data are normalized to protein concentration and are shown as mean ± SEM (*n* = 4 for receiver cells grown in control medium, *n* = 5 for receiver cells grown in post-therapy medium). *p < 0.05, **p < 0.01, ***p < 0.001 (Student’s *t-*test) where indicated. The experiments were replicated twice with similar results. Data from one set of experiments are shown.

**Figure 3.**
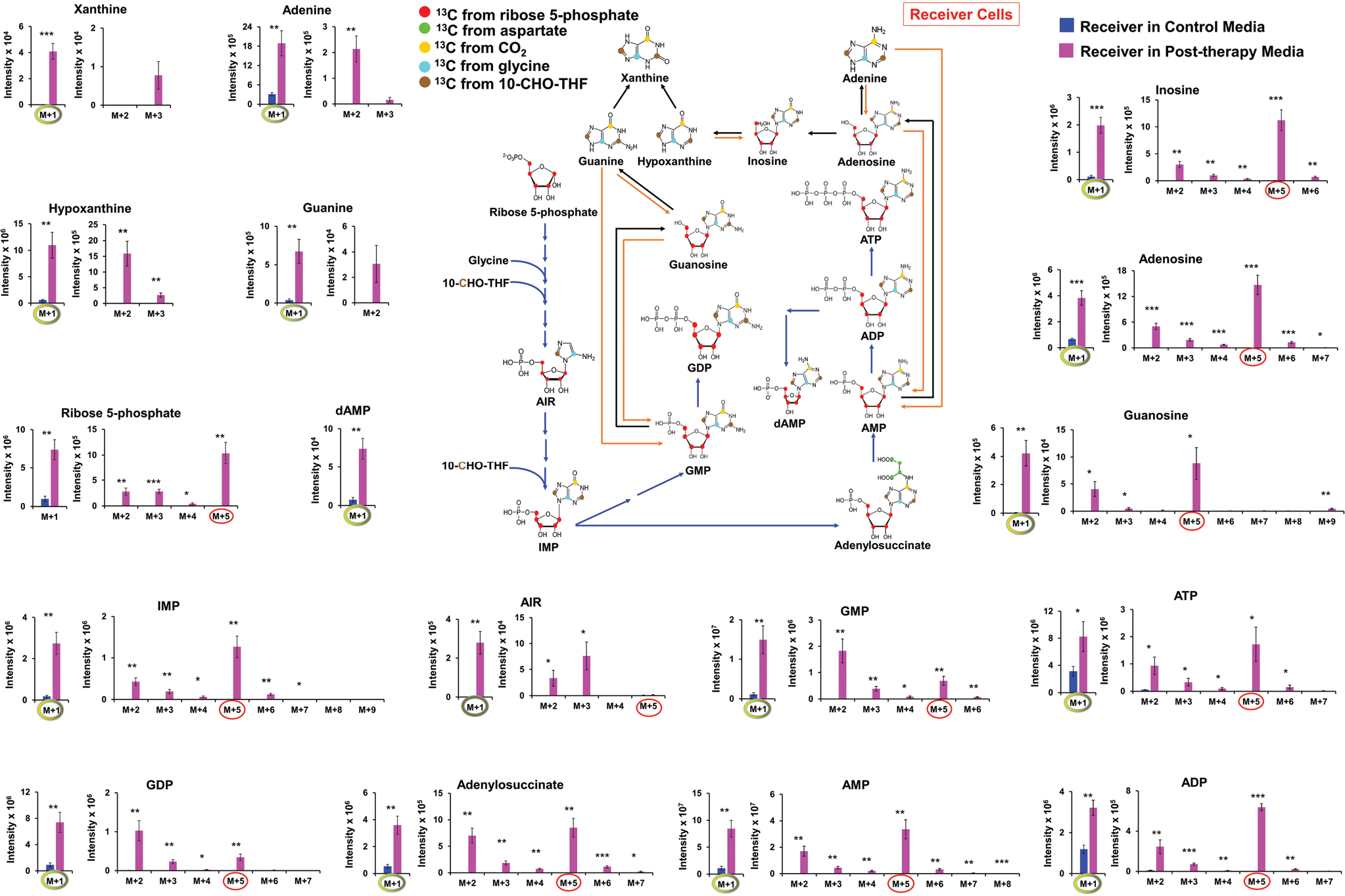
Isotopologues of ^13^C-labeled purine metabolites of receiver cells grown in post-therapy medium or in control medium and illustration of the corresponding purine metabolism pathways. Metabolite intensities of each isotopologue of receiver cells grown in post-therapy medium containing the dead donor cell debris (previously grown with ^13^C-labeled glucose) for 72 h are shown as pink bars; metabolite intensities of each isotopologue of receiver cells grown in control medium are shown as blue bars. Metabolites are shown in black. Red dots represent labeled ^13^C from ribose 5-phosphate. Green dots represent labeled ^13^C from aspartate. Yellow dots represent labeled ^13^C from CO_2_. Light blue dots represent ^13^C from glycine. Brown dots represent ^13^C from 10-CHO-THF. Blue arrows indicate de novo synthesis, black arrows indicate catabolism, and orange arrows indicate salvage of the purine metabolites. The (m+5) isotopologues shown in red circles originate most likely from (m+5) ribose 5-phosphate. The (m+1) isotopologues shown in yellow to brown gradient circles with light blue outlines originate most likely from (m+1) glycine, (m+1) 10-CHO-THF, or (m+1) CO_2_. Data are normalized to protein concentration and are shown as mean ± SEM (*n* = 4 for receiver cells grown in control medium, *n* = 5 for receiver cells grown in post-therapy medium). *p < 0.05, **p < 0.01, ***p < 0.001 (Student’s *t-*test) where indicated. The experiments were replicated twice with similar results. Data from one set of experiments are shown.

**Figure 4.**
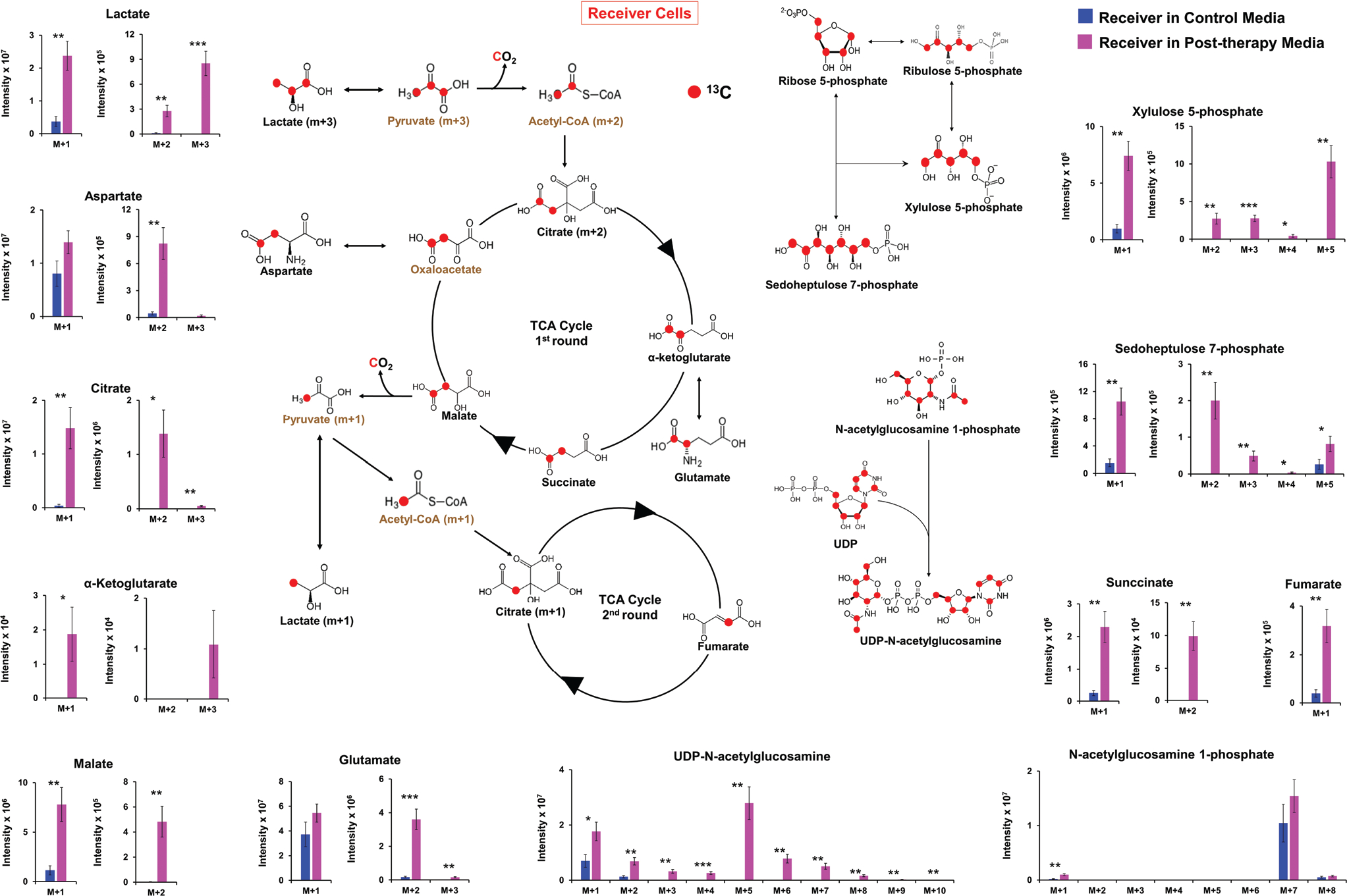
Isotopologues of ^13^C-labeled carbohydrate and hexosamine pathway metabolites of receiver cells grown in post-therapy medium or in control medium and illustration of the corresponding pathways. Metabolite intensities of each isotopologue of receiver cells grown in post-therapy medium containing the dead donor cell debris (previously grown with ^13^C-labeled glucose) for 72 h are shown as pink bars; metabolite intensities of each isotopologue of receiver cells grown in control medium are shown as blue bars. Metabolites found in receiver cells are shown in black. Red dots represent labeled ^13^C. Data are normalized to protein concentration and are shown as mean ± SEM (*n* = 4 for receiver cells grown in control medium, *n* = 5 for receiver cells grown in post-therapy medium). *p < 0.05, **p < 0.01, ***p < 0.001 (Student’s *t-*test) where indicated. The experiments were replicated twice with similar results. Data from one set of experiments are shown.

The growth-promoting effects of the post-therapy environment we observed in vitro and in vivo led us to investigate *how* this specific environment increased the proliferation of cancer cells beyond their intrinsic fast-growing characteristics. We hypothesize that dead donor cell debris had served as a nutritional source of “off-the-shelf” and precursor metabolites that receiver cells could benefit from. Since the post-therapy medium enabled receiver cells to proliferate in glucose-free medium, we suspected that part of the “off-the-shelf” and precursor metabolites released from dead donor cells could be products made from glucose originating from dead donor cells. We grew donor cells in a ^13^C_6_-glucose medium for 2 days, and then in the non-labeled medium after a thorough washing, followed by UV radiation treatment. Receiver cells were then cultured in this post-therapy medium using the transwell system as described above, where dead donor cells were the only available source capable of releasing labeled ^13^C metabolites. We assessed all the ^13^C-labeled metabolites in receiver cells after 3 days of growth in control or post-therapy medium (Figure 1A-δ) and found that all detectable isotopologues of ^13^C-labeled metabolites were exclusively in receiver cells grown in post-therapy medium (Figures 2–4). The detectable ^13^C-labeled metabolites in receiver cells grown in the control medium were (m+1) isotopologues, which were at overall lower intensities as compared to the isotopologues found in receiver cells grown in post-therapy medium. (M+1) isotopologues are also considered endogenous labeled as reported.^[17]^ The presence of all isotopologues of ^13^C-labeled metabolites beyond (m+1) in receiver cells grown in a post-therapy medium (but not in control medium) demonstrates that these labeled metabolites resulted from the dead donor cells. Of note, the metabolic profile of the samples is a snapshot taken at the moment the samples were harvested, which reflects the current metabolic process at that instant. Thus, we performed the experiments at different time points (24, 48, 72 h) and obtained similar results for each time point (however, only data for the 72 h time point are presented here). The intensities presented in the graphs reflect the number of ions detected from a given compound. The majority of ^13^C-labeled metabolites found are involved in nucleotide metabolism, which includes pyrimidine (Figure 2, Figure S1, Supporting Information, Table 1) and purine metabolism (Figure 3, Figure S2, Supporting Information, Table 1), as well as carbohydrate and amino acid metabolism (Figure 4, Figures S3–S5, Supporting Information, Table 1). Labeled metabolites detected in receiver cells could be a result of two processes: either they were directly absorbed as “off-the-shelf” metabolites from the post-therapy medium, or they were synthesized by receiver cells using ^13^C from precursor metabolites taken up from the medium.

To identify which metabolites were “off-the-shelf” and which were newly synthesized, the post-therapy medium alone (without receiver cells) was also analyzed to ensure that the collected metabolites originated solely from dead donor cells and did not include contributions from receiver cells (Figure 1A-β). Furthermore, we also sought to compare the isotopic distribution patterns (% labeled-isotope) of the post-therapy medium alone with those of receiver cells. We found several key ^13^C-labeled metabolites involving de novo and salvage pyrimidine synthesis in receiver cells in which labeled aspartate, ribose-5-phosphate, uridine, and uracil were also present in the post-therapy medium (Figure S1A,B, Supporting Information), suggesting that these metabolites could be directly absorbed by receiver cells from the post-therapy medium. In receiver cells, ^13^C-labeled aspartate together with ^13^C-labeled ribose-5-phosphate participated in de novo pyrimidine synthesis to make ^13^C-labeled pyrimidine metabolites, including uridine monophosphate (UMP), uridine diphosphate (UDP), cytidine monophosphate (CMP), cytidine diphosphate (CDP), and cytidine triphosphate (CTP) (Figure S1B, Supporting Information, metabolites in black). We also found deoxyribonucleotides such as deoxycytidine monophosphate (dCMP), deoxycytidine diphosphate (dCDP), thymidine monophosphate (TMP), and thymidine diphosphate (TDP) (Figure S1B, Supporting Information, metabolites in light blue). The presence of labeled UMP, UDP, (d)CMP, (d)CDP, CTP, TMP, TDP could be explained by a contribution of (m+1 to 5) ribose-5-phosphate and labeled (m+1) CO_2_, and/or (m+1, 2, 3) aspartate resulting from the de novo pyrimidine synthesis pathway (Figure S1B, Supporting Information, blue arrows). Moreover, for UMP, UDP, CMP, CDP, and CTP, the (m+5) isotopologues were found to be predominant in receiver cells, suggesting a high degree of incorporation of the labeled 5-carbon ring ribose skeleton (Figure S1B, Supporting Information). The presence of labeled UMP, UDP, (d)CMP, (d)CDP, CTP, TMP, TDP could also be explained by the direct “off-the-shelf” labeled uridine, ribose-5-phosphate, and uracil in the medium being transferred to receiver cells during pyrimidine synthesis via the salvage pathway (Figure S1B, Supporting Information, orange arrows). While labeled-uracil and uridine were present in both post-therapy medium and receiver cells with similar isotopic distribution patterns as “off-the-shelf” metabolites of the pyrimidine salvage pathway in receiver cells (Figure S1A,B, Supporting Information), the presence of labeled-cytidine in receiver cells alone strongly indicates that the receiver cells underwent pyrimidine catabolism from labeled-CMP (Figure S1B, Supporting Information, black arrows). After confirming the presence of ^13^C-labeled pyrimidine metabolites in receiver cells grown in the post-therapy medium, we assessed non-labeled (m+0) intensities of these pyrimidine metabolites in receiver cells grown in post-therapy and control medium to investigate how post-therapy medium altered the overall pyrimidine metabolic profile in receiver cells. We found that the intensities of pyrimidine metabolites, such as ribose-5-phosphate, uracil, uridine, UDP, TMP, TDP, cytidine, cytosine, CTP, CDP, (d)CMP, were all between 3 and 42 times higher in receivers cells grown in the post-therapy medium with UV-irradiated donor cells as compared to those grown in the control medium without UV-irradiated donor cells (Figure S6, Supporting Information).

The assessment of all the ^13^C-labeled metabolites in receiver cells grown in post-therapy medium containing UV-irradiated donor cells also showed ^13^C-labeled metabolites involved in purine synthesis (Figure 3), in which labeled-guanine and hypoxanthine were also present in the post-therapy medium (Figure S2A,B, Supporting Information), suggesting that these metabolites could be directly absorbed by receiver cells from the post-therapy medium. The presence of ^13^C-labeled-aminoimidazole ribotide (AIR) and adenylosuccinate strongly indicates that de novo purine synthesis occurred using labeled compounds taken up from the post-therapy medium. The labeled-inosine monophosphate (IMP), adenylosuccinate, guanosine monophosphate (GMP), guanosine diphosphate (GDP), adenosine monophosphate (AMP), adenosine diphosphate (ADP), and adenosine triphosphate (ATP) could be explained by a contribution of labeled-ribose-5-phosphate and labeled (m+1) CO_2_ or (m+1) glycine or (m+1) 10-formyltetrahydrofolate (10-CHO-THF) in the process of de novo purine synthesis (Figure S2B, Supporting Information, blue arrows). The presence of (m+1) isotopologues at the highest peak intensity among all the isotopologues in every metabolite found in purine metabolism in both the post-therapy medium and in receiver cells could be explained by the high incorporation of components of the one-carbon metabolism pathway, or several rounds of metabolic processing (Figure S2A,B, Supporting Information). The presence of labeled IMP, GMP, GDP, AMP, ADP, and ATP could also be explained by the contribution of “off-the-shelf” (m+1, 2) guanine or (m+1, 2, 3) hypoxanthine in the medium being transferred to the receiver cells and ribose-5-phosphate in the process of the purine salvage pathway (Figure S2B, Supporting Information, orange arrows). While labeled-guanine and hypoxanthine were present in both post-therapy medium and receiver cells with similar isotopic distribution patterns as “off-the-shelf” metabolites for the purine salvage pathway in receiver cells (Figure S2A,B, Supporting Information), the presence of labeled-adenosine, adenine, guanosine, and inosine in receiver cells, but not in post-therapy medium, strongly indicates that the receiver cells also underwent purine catabolism from labeled-AMP, GMP, and IMP (Figure S2B, Supporting Information, black arrows). Similar to the strategy we used in the previous section for studying pyrimidine metabolism, after confirming the presence of ^13^C-labeled purine metabolites in receiver cells grown in the post-therapy medium, we assessed non-labeled (m+0) intensities of these purine metabolites in receiver cells grown in post-therapy and control medium to investigate how the post-therapy medium altered the overall purine metabolic profile in receiver cells. The results showed that similar to pyrimidine metabolism, the intensities of purine metabolites, such as AIR, IMP, GMP, GDP, guanosine, guanine, hypoxanthine, adenylosuccinate, (d)AMP, ADP, ATP, inosine, adenosine, adenine, and xanthine, were all between 1.5 and 16 times higher in receivers cells grown in the post-therapy medium as compared to those grown in the control medium (Figure S7, Supporting Information).

Lastly, we observed, in receiver cells, the presence of ^13^C-label in amino acids (aspartate, alanine, glutamate, O-acetylserine, proline, and arginine) (Figures S4 and S5, Supporting Information), as well as in carbohydrate metabolites, including the final product of glycolysis (lactate), intermediates of the tricarboxylic acid (TCA) cycle (citrate, α-ketoglutarate, succinate, fumarate, and malate) (Figure 4 and Figure S3, Supporting Information), and intermediates of the pentose phosphate pathways (xylulose-5-phosphate and sedoheptulose-7-phosphate) and hexosamine pathway (*N*-acetylglucosamine 1-phosphate and UDP-*N*-acetylglucosamine) (Figure 4 and Figure S3, Supporting Information). Labeled lactate, aspartate, sedoheptulose-7-phosphate, glutamate, and proline were also present in the post-therapy medium (Figures S3A and S5A, Supporting Information). The (m+1) isotopologue has the highest intensity among all the isotopologues (Figures S3B and S5B, Supporting Information). The accumulation of (m+1) isotopologues in post-therapy medium and receiver cells could be due to several rounds of metabolic processing in donor cells, and then in receiver cells after labeled metabolites were absorbed from the post-therapy medium (Figure S3, Supporting Information). The post-therapy medium also contains high intensities of (m+3) lactate, (m+2) glutamate, and (m+2) aspartate, which are direct products of (m+6) glucose metabolism in donor cells (Figure S3A, Supporting Information). Assessment of non-labeled metabolites in receiver cells revealed that the intensities of lactate, TCA cycle metabolites (citrate, malate, succinate, fumarate, α-ketoglutarate), amino acids (glutamine, serine, arginine, tyrosine, O-acetylserine, isoleucine, leucine, methionine, phenylalanine, proline, tryptophan, tyrosine, and histidine), and metabolites from the pentose phosphate pathway (xylulose-5-phosphate and sedoheptulose-7-phosphate) and the hexosamine pathway (*N*-acetylglucosamine 1-phosphate and UDP-*N*-acetylglucosamine) were between 1.3 and 43 times higher in receiver cells grown in the post-therapy medium as compared to those grown in the control medium (Figure S8, Supporting Information). Importantly, the higher intensities of essential amino acids (isoleucine, leucine, methionine, phenylalanine, proline, tryptophan, and histidine) in receiver cells grown in the post-therapy medium as compared to those grown in the control medium again indicate that the dead donor cells released these essential amino acids from endogenous pools into the post-therapy medium after UV irradiation, creating an environment with higher essential amino acid concentrations as compared to the control medium since essential amino acids cannot be made de novo in the receiver cells.

To investigate whether the active uptake of metabolites from the post-therapy medium is an oncogenic behavior, we performed all the labeled and non-labeled experiments mentioned above, except that we used *MYC*-OFF P493 cells. First, there were no traces of ^13^C-labeled metabolites detected in *MYC*-OFF receiver cells beyond the endogenous (m+1) isotopologue (Figure S9A, Supporting Information). Moreover, there were no significant differences in the overall peak intensities of pyrimidine, purine, carbohydrate, and amino acid metabolites between receivers cells grown in the post-therapy medium as compared to those grown in the control medium (Figure S9B, Supporting Information). These findings indicate that *MYC*-OFF receiver cells did not actively absorb more metabolites from the post-therapy medium as compared to the control medium. While it is possible that non-oncogenic cells can also scavenge metabolites from the post-therapy medium, one possible explanation for these findings could be that the *MYC*-OFF cells are not proliferating (Figure 1E) and thus potentially have reduced metabolic activity. The result thus reveals a clear distinction in metabolic behaviors of oncogenic versus non-oncogenic cells and serves as proof that the uptake of metabolites from a post-therapy environment is a result of aggressive carcinogenic behavior. It is important to note that *MYC* expression has previously been shown to reprogram and promote many aspects of cell metabolism including glucose, glutamine, proline, and nucleotide metabolism.^[7,18]^

Several previous studies have revealed the transfer of materials from *live* cancer-associated fibroblasts (CAFs) to neighboring cancer cells through either exosomes or direct metabolite release into the tumor microenvironment.^[19–22]^ The uptake of these CAF-derived exosomes (CDEs) or metabolites has been shown to promote the proliferation of pancreatic and prostate cancer cells.^[19–22]^ Specifically, the CDEs altered many vital metabolic pathways of the cancer cells, including glucose metabolism, glutamine metabolism, fatty acid metabolism, and amino acid metabolism, thus promoting survival and proliferation.^[19,20]^ Inhibiting the transfer of materials from CAFs to surrounding cancer cells showed a reduction in cancer growth.^[21,22]^ Another set of studies showed that hypoxia and oxidative stress caused by either neighboring cancer cells or pharmacological treatment could induce autophagy in CAFs, which then provides the adjacent cancer cells with metabolites such as lactate, amino acids, and nucleotides that promote proliferation.^[23,24]^ However, of distinction, in the current study, we used *MYC*-ON P493 cells *without CAFs* and provide strong evidence for the transfer of metabolites released from the *dead* to live cancer cells. A few studies have also previously demonstrated the transfer of genetic materials from dead or dying cancer cells to living cancer cells.^[25,26]^ Another study has shown that macropinocytosis of protein is an amino acid supply route in Ras-transformed cells.^[27]^ Similarly, another study of prostate cancer has shown that the cells are capable of using the proteins and lipids in the necrotic cell debris obtained via macropinocytosis for synthesizing new proteins and maintaining lipids.^[28]^ However, none of these studies have demonstrated that the live cancer cells can take up metabolites directly. Therefore, the current study reveals, for the first time, that surviving cancer cells “*inherit”* metabolic materials from *dead* cancer cells post-therapy and not just from live CAFs. This helps increase their proliferation in vitro and probably in vivo. Importantly, this “metabolic recycling” phenomenon only occurs in oncogenic cells.

## 3. Conclusion

To summarize, in this short report, we showed that the readily available metabolites in a post-therapy environment are utilized by living cancer cells to proliferate. We identified in detail 1) that cancer cells accumulate metabolic materials from *dead* cancer cells post-therapy as nutritional sources of “off-the-shelf” and precursor metabolites and 2) that these metabolites enter into multiple vital metabolic processes (namely, those associated with carbohydrate, amino acid, and nucleotide metabolism). This incorporation leads to the overall metabolite peak intensities being between 1.3 and 43 times higher in cancer cells grown in a post-therapy medium compared to those grown in control medium, resulting in significantly increased growth in vitro and probably in vivo. The extensive presence of ^13^C-labeled metabolites in receiver cells and the evidence that they are further integrated into multiple metabolic processes in these cells reveals that vital metabolites released by *dead* cells post-therapy are re-used by receiver cells, a phenomenon that we have named “metabolic recycling” of oncogenic cells. This metabolic behavior of cancer cells could explain in part the fast re-proliferation and tumorigenesis ability of surviving cancer cells post-therapy, paving the way for new strategies to prevent cancer relapse. Because metabolic recycling is not the sole contributing factor to cancer relapse, further investigations are needed to achieve successful clinical applications. Further studies on cell signaling pathways and specific metabolic transporters for this metabolic recycling phenomenon should be investigated to obtain more details underlying the recurrence of cancer. Also, we have not explored this finding on other oncogene models, which will be the focus of our future work. Future research should also aim to confirm and further investigate the observation and mechanism of the metabolic recycling phenomenon in vivo. The use of inhibitory drugs to decrease the uptake of metabolites by cancer cells from the post-therapy environment could serve as potential treatments for cancer patients to decrease cancer recurrence rates. Since the metabolic recycling behavior was only observed in oncogenic cells (specifically, *MYC*-ON cells in this study), a therapeutic approach interrupting this process could target cancer cells only and spare normal cells. *However, the present work shows for the first time that metabolites derived from dead cancer cells may be utilized by surviving cancer cells to their proliferative advantage*. The identification of the growth-promoting effect caused by the post-therapy environment should encourage further genetic, proteomics, and metabolomics studies to form a coherent and comprehensive picture of cancer cell behavior post-therapy.

## 4. Experimental Section

### Reagents: Cell Lines:

The P493 human Burkitt lymphoma B cells were provided and authenticated by Dr. Chi V. Dang’s laboratory (Wistar Institute).^[6]^ The P493 cells were grown in Roswell Park Memorial Institute medium (RPMI) supplemented with 10% vol/vol fetal bovine serum (FBS), 1% vol/vol penicillin-streptomycin (full RPMI) prior to experimental analysis. Cells were incubated at 37 °C in the presence of 5% vol/vol CO_2_.

### Mice and Mice Housing:

The animal study protocol complied with the Association for Assessment and Accreditation of Laboratory Animal Care guidelines and was approved by Johns Hopkins University Animal Care and Use Committee (MO18M42I). Male 4 weeks old NCI SCID C.B-17/Icr mice (Charles River) weighing 22–24 g were used for the in vivo study. These SCID mice also had the following characteristics: albino coat, auto-recessive, single-nucleotide polymorphism within the Prkdc gene on chromosome 16, and severe combined immunodeficiency affecting T- and B-cell development. Natural Killer (NK) cell, macrophage, and granulocyte were normal as per the manufacturer’s information.

### Determination of UV Dose Needed to Completely Eradicate P493 Donor Cells In Vitro:

The UV radiation dose used to eradicate all donor cells was determined by testing various doses of UV radiation on live P493 cells seeded into 6-well transwell plates or 96-well plates (for cell and tumor growth evaluation) or a 10cm transwell dish (for metabolic analysis). P493 donor cells were grown at a confluency of 100 000 cells mL^−1^ in normal full RPMI prior to UV irradiation. For cell growth evaluation, 1 × 10^6^ P493 donor cells in 500 μL of full RPMI were seeded onto the bottom compartment of each well of the 6-well transwell plates (Corning). For tumor growth evaluation, 50 × 10^6^ P493 donor cells in 150 μL of RPMI were seeded onto each well of a 96 well plate. For metabolic analysis, 45 × 10^6^ P493 donor cells in 3 mL full RPMI with dialyzed FBS were seeded onto the bottom compartment of 10 cm transwell dishes (Corning). The cells were subjected to a variety of UV-doses generated by a UV Translinker TL-2000 and were then cultured at 37 °C in the presence of 5% vol/vol CO_2_. The viability of the cells was evaluated daily post-UV irradiation using a Vi-Cell XR Cell Viability Analyzer (Beckman Coulter). The selected UV-dose (20 000 J m^−2^ for 1 × 10^6^ P493 donor cells and 80 000 J m^−2^ for 45 × 10^6^ or 50 × 10^6^ P493 donor cells) was determined as the minimum dose of UV needed to eradicate all P493 donor cells at every time point evaluated.

### Culture of P493 Cells in Post-Therapy Medium and Control Medium In Vitro for Cell Growth Evaluation:

For the post-therapy medium, 1 × 10^6^ P493 cells (donor cells) in 500 μL full RPMI medium were seeded onto the bottom compartment of the top wells of 6-well transwell plates (Corning). For the control medium, 500 μL of cell-free full RPMI medium were placed onto the bottom compartment of bottom wells of the same 6-well transwell plates. The plates were then subjected to 20 000 J m^−2^ of UV radiation (dose determined as described above). Following UV irradiation, 2.1 mL non-irradiated full RPMI medium was added to each well. The top compartment, which possessed a membrane with a 0.4 μm pore size, was then inserted into each well. The membrane of the top compartment prevented the P493 cells (diameter: 12–14 μm)^[13]^ from traveling between compartments. 2 × 10^4^ live P493 receiver cells were seeded in the top compartment with 1.5 mL of full RPMI, maintaining the co-culturing ratio at 1 receiver cell (top compartment) to 50 donor cells (bottom compartment). Receiver cells were then cultured at 37 °C with 5% vol/vol CO_2_. Cell number and viability were assessed every 3 days using a Vi-Cell XR Cell Viability Analyzer for a total of 12 days. For glucose-free experiments, all the procedures were the same except that the full RPMI medium was replaced with full RPMI without glucose.

### Tetracycline Treatment to Suppress the MYC Oncogene:

Prior to the cell growth evaluation and metabolic analysis experiments, *MYC-*ON P493 cells, which have a tetracycline-repressible *MYC* construct, were treated with tetracycline at a concentration of 0.1 μg mL^−1^ to suppress the *MYC* oncogene as previously described.^[2–7]^ All the experiments were also performed in the same manner as described for the *MYC*-ON cells in vitro.

### Culture of P493 Cells in Post-Therapy Medium and Control Medium In Vivo for Tumor Growth Evaluation:

5 × 10^7^ P493 cells (donor cells) in 150 μL RPMI were plated onto each well (*n* = 40) of a 96 well plate. 150 μL of cell-free RPMI was plated into each well (*n* = 20) of the same 96-well plate. The plate was then irradiated with a UV dose of 80 000 J m^−2^ to kill all the cells. To investigate whether post-therapy media containing dead donor cells were able to promote tumorigenesis, post-therapy media containing dead donor cells were harvested (*n* = 20) after UV irradiation and injected into five SCID mice, each at four sites in the flank. These mice were then monitored for 7 weeks to confirm that no tumors were formed. Simultaneously, the viability of donor cells (if any) after UV irradiation from the remaining wells (*n* = 20) were assessed. [Of note, the hair on the back of the mice was completely removed on the day of injection. However, the hair grew back heterogenously throughout the course of the experiment.] Next, to evaluate the tumor growth-promoting effect of post-therapy medium containing dead donor cell debris, 1 × 10^6^ live P493 receiver cells in 50 μL RPMI were added into each well of post-therapy medium containing dead donor cells or control medium after UV irradiation and thoroughly mixed. Receiver cells in each well were aspirated into a 1 mL syringe and injected subcutaneously into five SCID mice, each at four sites in the flank. Tumor sizes were measured at day 13, 25, and 37 days post-injection.

### Culture of P493 Cells in Post-Therapy Medium and Control Medium In Vitro for Metabolic Analysis:

P493 donor cells were grown at a confluency of 100 000 cells mL^−1^ in full RPMI, except that normal ^12^C_6_-glucose was replaced by uniformly fully labeled ^13^C_6_-glucose (m+6 glucose) for 48 h. On the day of culture in the transwell apparatus, the medium containing ^13^C_6_-glucose was removed, and the donor cells were thoroughly washed with phosphate-buffered saline (PBS). 4.5 × 10^7^ P493 donor cells in 3 mL non-labeled full RPMI were then seeded onto the bottom compartment of each 10 cm transwell dish and subjected to UV radiation at a dose of 80 000 J m^−2^ to completely eradicate all live P493 cells in the dish. 3 mL non-labeled full RPMI medium was also placed onto the bottom compartment of each 10 cm transwell dish and subjected to the same dose of UV radiation as the control medium. After UV irradiation, 10 mL of non-irradiated non-labeled standard full RPMI medium was added to each dish. The top compartment, which possessed a membrane with 0.4 μm pore size, was then inserted into each dish, in which 0.9 × 10^6^ live P493 receiver cells in 9 mL of normal non-labeled full RPMI were seeded, maintaining the co-culturing ratio of 1 receiver cell (top compartment): 50 donor cells (bottom compartment). The receiver cells were grown in a post-therapy medium containing dead donor cell debris or control medium receiving the same UV-dose, for 24, 48, and 72 h. These two groups were used to identify ^13^C-labeled metabolites released by dead donor cells into the post-therapy medium and taken up by receiver cells as compared to those grown in the control medium. Two other groups of receiver cells were co-cultured in post-therapy medium containing dead donor cell debris or control medium in a similar manner to that mentioned above, except that the donor cells were grown in full RPMI with ^12^C_6_-glucose (non-labeled) instead of ^13^C_6_-glucose. These two groups enabled the overall metabolic profiling of receiver cells in the post-therapy medium. Receiver cells were harvested after 24, 48, and 72 h and subjected to metabolic extraction and analysis.

The entire procedure was repeated with *MYC*-OFF P493 cells to compare the metabolic behavior between oncogenic and nononcogenic cells.

### Culture of P493 Cells In Vitro for Metabolic Analysis of Post-Therapy Medium:

To identify which metabolites were “off-the-shelf” and which were newly synthesized, the post-therapy medium alone (without receiver cells) was also analyzed to ensure that the collected metabolites solely originated from dead donor cells and did not include contributions from receiver cells. Specifically, P493 donor cells were grown at a confluency of 100 000 cells mL^−1^ in full RPMI in which normal ^12^C_6_-glucose was replaced by uniformly fully labeled ^13^C_6_-glucose (m+6 glucose) for 48 h. On the day of UV irradiation, the medium containing ^13^C_6_-glucose was removed, and the donor cells were thoroughly washed with PBS. 45 × 10^6^ P493 donor cells in 3 mL non-labeled full RPMI were then seeded onto the bottom compartment of each 10 cm transwell dish and subjected to UV irradiation at a dose of 80 000 J m^−2^ to completely eradicate all live P493 cells in the dish. 3 mL non-labeled full RPMI was also placed onto the bottom compartment of each 10 cm transwell dish and subjected to the same dose of UV radiation to serve as a control medium. After UV irradiation, 10 mL of non-irradiated, non-labeled normal full RPMI were added to each dish. 1 mL of the post-therapy medium was subjected immediately, and at 24, 48, and 72 h post-UV irradiation, to metabolic extraction and analysis.

### Metabolic Extraction, Data Acquisition, and Data Analysis:

Harvested receiver cells were centrifuged at 433 g for 10 min and washed with PBS. The supernatant was then removed, and the cells were snap-frozen in liquid nitrogen. To enable maximum and efficient metabolite extraction, the frozen cell pellets were subjected to metabolite extraction using cold 80% vol/vol liquid chromatography–mass spectrometry (LC-MS) grade methanol diluted with mass-spec-grade water as previously described.^[2,29,30]^ Similarly, the medium was collected after centrifugation and snap-frozen in liquid nitrogen. The frozen medium was then subjected to metabolite extraction by adding 100% LC-MS grade methanol to a final concentration of 80% vol/vol methanol. The samples were subjected to Speed-vac and were then lyophilized to remove the methanol–water mixture. Dried metabolites were then re-suspended in 50% vol/vol acetonitrile diluted with MS water. Metabolomic data from these samples were acquired using a Thermo Scientific Q Exactive Plus Orbitrap Mass Spectrometer coupled with a Vanquish UPLC system at Metabolomics Facility at Johns Hopkins Medical Institute.

The Vanquish UPLC autosampler was used to withdraw and inject 2 μL of the sample into the LC/MS system for data acquisition. The samples were maintained at 4 °C during acquisition in the autosampler chamber to ensure a stable environment. Reverse-phase chromatography was used in which the mobile aqueous phase was 0.1% formic acid in MS grade water, and the mobile organic phase was 0.1% formic acid in acetonitrile. A Discovery HSF5 reverse phase high-performance liquid chromatography column (Sigma) was used for acquisition. The total run time was 13 min where 11 min was dedicated to data acquisition and 2 min post-acquisition was used for re-equilibration of the chromatographic column, which was also equipped with a guard column, maintained at 35 °C. Prior to every data acquisition, a mass calibration was performed to ensure sensitivity and accuracy of data acquired. Full MS scans were acquired for quantification of metabolites in samples along with full MS/ddMS2 scans to enable identification of metabolites via fragmentation matching.

Data were then analyzed using Thermo Scientific Compound Discoverer software for qualitative and quantitative analysis of non-labeled metabolites. The specific isotopologues of metabolites were detected using Compound Discoverer and later quantified using Xcalibur Processing Setup and Quan browser and Thermo Scientific TraceFinder. The chromatographic peaks were integrated to obtain raw intensities of metabolites, which were then normalized to the protein concentration of each sample to obtain the final normalized intensities.

### Protein Concentration Measurement:

Protein from each sample was extracted using M-PER Mammalian Protein Extraction Reagent with added protease inhibitor. Protein concentration was then measured using the Pierce BCA Protein Assay Kit.

### Statistical Analysis:

The intensities presented were the chromatogram peak areas which corresponded to the number of ions detected for the compounds after normalization to protein concentration of each sample. The % labeled-isotope of each labeled isotopologue was calculated by dividing the normalized intensity of each labeled isotopologue by the total intensity of all labeled isotopologues. No other pre-processing of data was performed. All data were shown as the mean ± SEM as described in the figure legends.^[2,18]^ Statistical details for the experiments, such as the number of biological replicates (*n*), could be found in the accompanying figure legends. Statistical significance was determined using one-tailed Student’s *t*-test with *p*-value < 0.05 considered significant and annotated as *p < 0.05, **p < 0.01, ***p < 0.001. All statistical analyses were performed using Microsoft Excel.

## Supplementary Material

Supinfo

## Figures and Tables

**Table 1.** Summary of labeled metabolites in receiver cells grown in post-therapy medium containing dead donor cells. The donor cells were incubated with ^13^C-labeled glucose and killed by UV irradiation. Then the receiver cells were grown in the post-therapy medium containing the dead donor cell debris for 72 h. The table is organized based on relevant metabolic pathways and whether the metabolite is “off-the-shelf” or de novo (newly synthesized in receiver cells) based on observed isotopic labeling pattern.

	“Off the shelf” metabolites	de novo metabolites

Pyrimidine metabolism	Aspartate	UMP
	Ribose 5-phosphate	UDP
	Uridine	TMP
	Uracil	TDP
		dCDP
		dCMP
		CTP
		CDP
		CMP
		Cytidine
Purine metabolism	Ribose 5-phosphate	AIR
	Guanine	IMP
	Hypoxanthine	GMP
		GDP
		Guanosine
		Adenylosuccinate
		AMP
		ADP
		ATP
		dAMP
		Adenosine
		Adenine
		Inosine
		Xanthine
Carbohydrate and hexosamine metabolism	Lactate	Citrate
	Aspartate	α-Ketoglutarate
	Glutamate	Succinate
	Ribose 5-phosphate	Fumarate
	Sedoheptulose 7-phosphate	Malate
		*N*-Acetylglucosamine
		1-phosphate
		UDP-*N*-acetylglucosamine
		Xylulose 5-phosphate
Amino acid metabolism	Lactate	Alanine
	Aspartate	Arginine
	Glutamate	O-Acetylserine
	Proline	

## Data Availability

The data that support the findings of this study are available from the corresponding author upon reasonable request.
