# Supplementary material for: Metabolic Recycling Enhances Proliferation in MYC-Transformed Lymphoma B Cells
Source: Adv Biol (Weinh). Author manuscript; Available in PMC 2024 Feb 1. (PMC10375452; doi:10.1002/adbi.202200233)
Supplement: Supinfo [file NIHMS1852867-supplement-Supinfo.pdf]

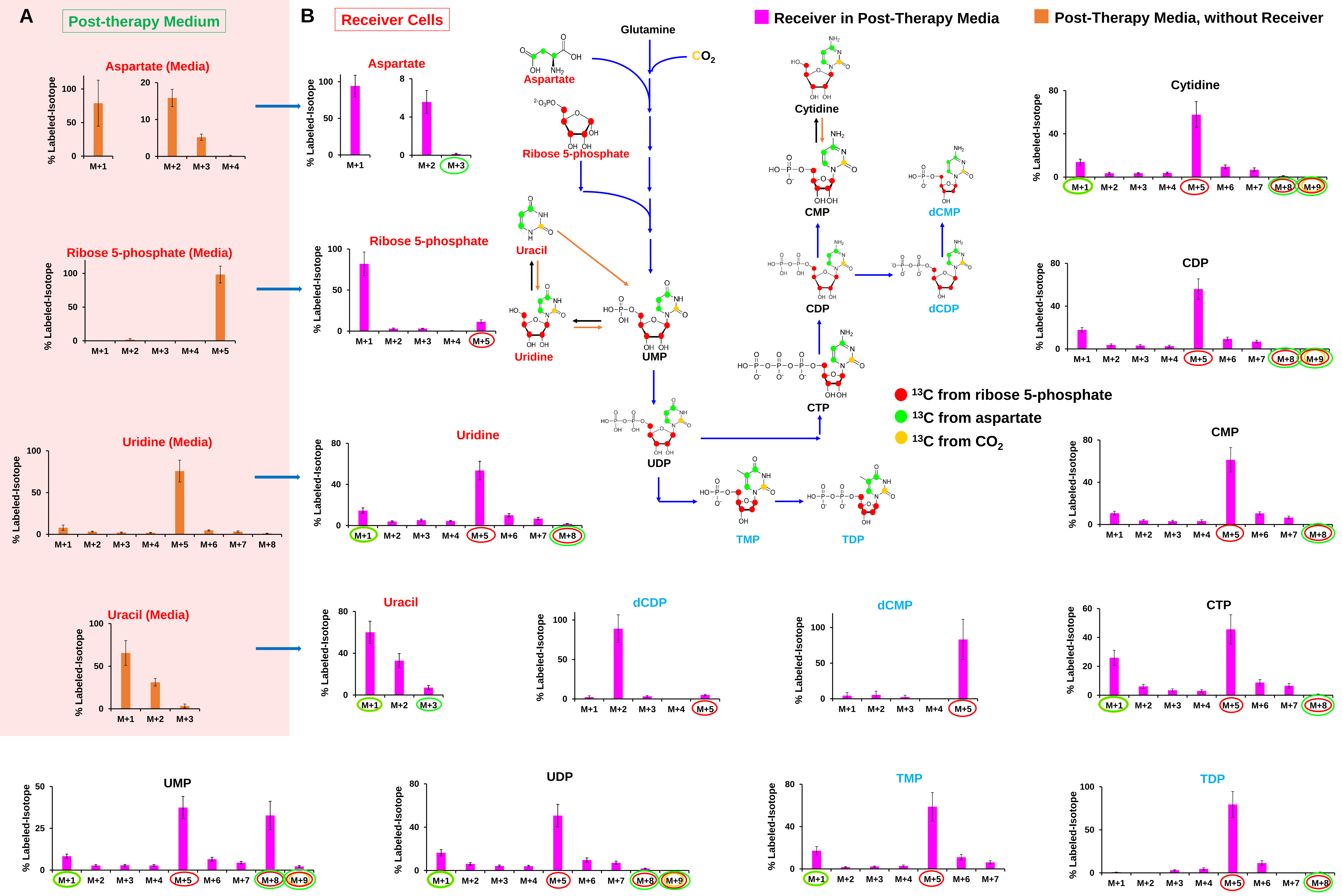

Figure S1. Percentage of ^{13}C-Labeled-Isotopes of Pyrimidine Metabolites in (A) Post-Therapy Medium Released from dead Donor Cells after UV Irradiation (B) Receiver Cells Grown in Post-Therapy Medium and Illustration of the Corresponding Pyrimidine Metabolism Pathways. Receiver cells were grown in post-therapy medium containing the dead Donor cell debris (previously grown with ^{13}C-labeled glucose) for 72 hours. Percentages of ^{13}C-labeled-isotope of metabolites found in post-therapy medium containing the dead Donor cell debris (previously grown with ^{13}C-labeled glucose) are shown as orange bars; percentages of ^{13}C-labeled-isotope of Receiver cells grown in post-therapy medium are shown as pink bars. Metabolites found in both the post-therapy medium and the Receiver cells (aspartate, ribose 5-phosphate, uracil, and uridine) are shown in red. Deoxyribonucleotide metabolites found only in the Receiver cells are shown in light blue. Other pyrimidine metabolites found only in the Receiver cells are shown in black. Red dots represent labeled ^{13}C from ribose 5-phosphate. Green dots represent labeled ^{13}C from aspartate. Yellow dots represent labeled ^{13}C from CO_2. Blue arrows indicate *de novo* synthesis, black arrows indicate catabolism, and orange arrows indicate salvage of the pyrimidine metabolites. The (m+5) isotopologues are shown in red circles. The (m+3) isotopologues are shown in green circles. The (m+1) isotopologues are shown in green circles with yellow outlines. The (m+8) isotopologues are shown as a combination of red and green circles. The (m+9) isotopologues are shown as a combination of red, green, and yellow circles. The % isotope enrichment was calculated by dividing the intensity of each isotopologue by intensities of total labeled isotopologues times 100%. Data are normalized to protein concentration and are shown as mean ± SEM (n = 4 for Receiver cells grown in control medium, n = 5 for Receiver cells grown in post-therapy medium). The experiments were replicated twice with similar results. Data from one set of experiments are shown.

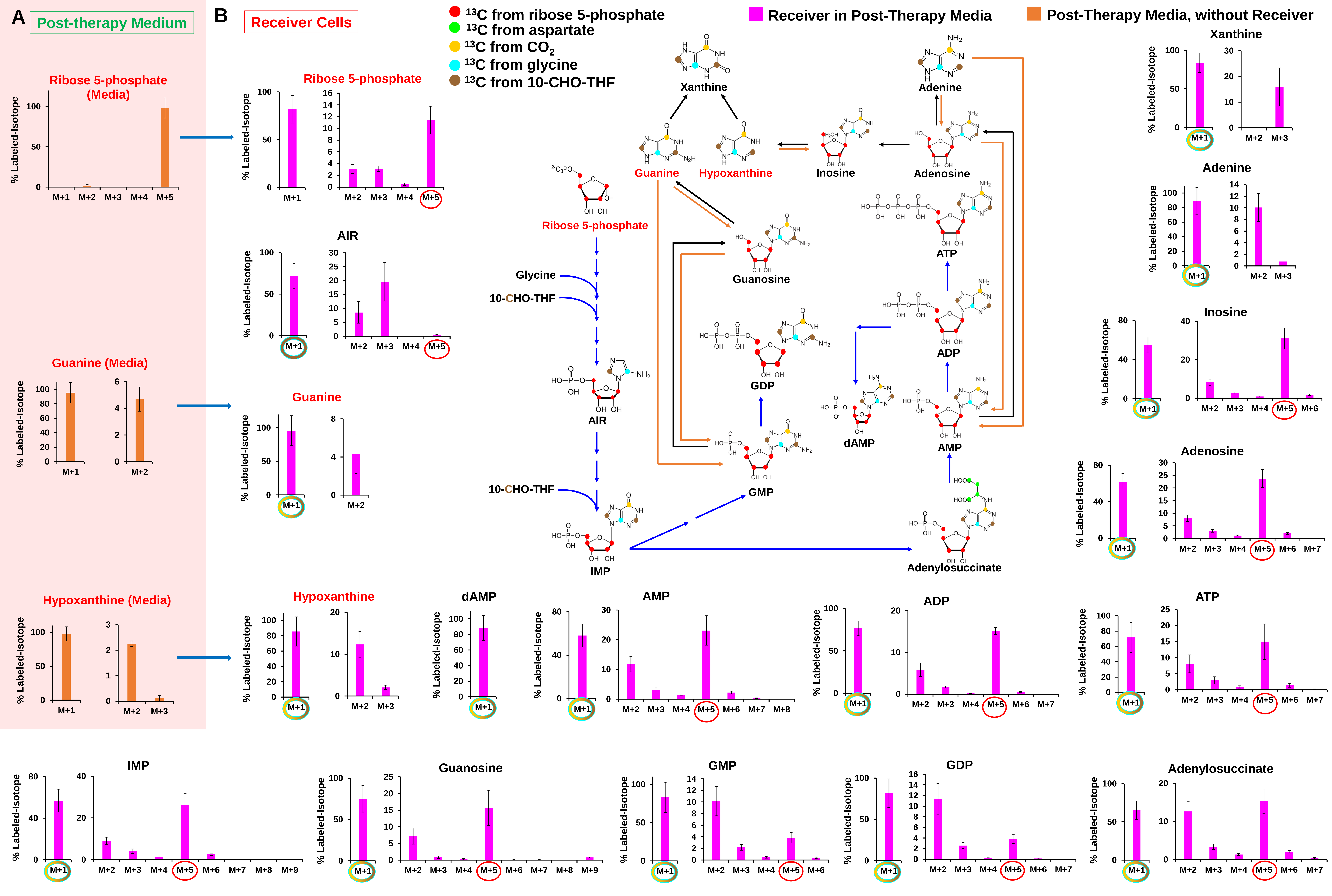

Figure S2. Percentage of ^{13}C-Labeled-Isotopes of Purine Metabolites in (A) Post-Therapy Medium Released from dead Donor Cells after UV Irradiation (B) Receiver Cells Grown in Post-Therapy Medium and Illustration of the Corresponding Purine Metabolism Pathways. Receiver cells were grown in post-therapy medium containing the dead Donor cell debris (previously grown with ^{13}C-labeled glucose) for 72 hours. Percentages of ^{13}C-labeled-isotope of metabolites found in post-therapy medium containing the dead Donor cell debris (previously grown with ^{13}C-labeled glucose) are shown as orange bars; percentages of ^{13}C-labeled-isotope of Receiver cells grown in post-therapy medium are shown as pink bars. Metabolites found in both the post-therapy medium and the Receiver cells (ribose 5-phosphate, guanine, xanthine, and hypoxanthine) are shown in red. Metabolites found only in the Receiver cells are shown in black. Red dots represent labeled ^{13}C from ribose 5-phosphate. Green dots represent labeled ^{13}C from aspartate. Yellow dots represent labeled ^{13}C from CO_2. Light blue dots represent ^{13}C from glycine. Brown dots represent ^{13}C from 10-CHO-THF. Blue arrows indicate *de novo* synthesis, black arrows indicate catabolism, and orange arrows indicate salvage of the purine metabolites. The m+5 isotopologues are shown in red circles. The (m+1) isotopologues are shown in yellow to brown gradient circles with light blue outlines. The % isotope enrichment was calculated by dividing the intensity of each isotopologue by intensities of total labeled isotopologues times 100%. Data are normalized to protein concentration and are shown as mean ± SEM (n = 4 for Receiver cells grown in control medium, n = 5 for Receiver cells grown in post-therapy medium). The experiments were replicated twice with similar results. Data from one set of experiments are shown.

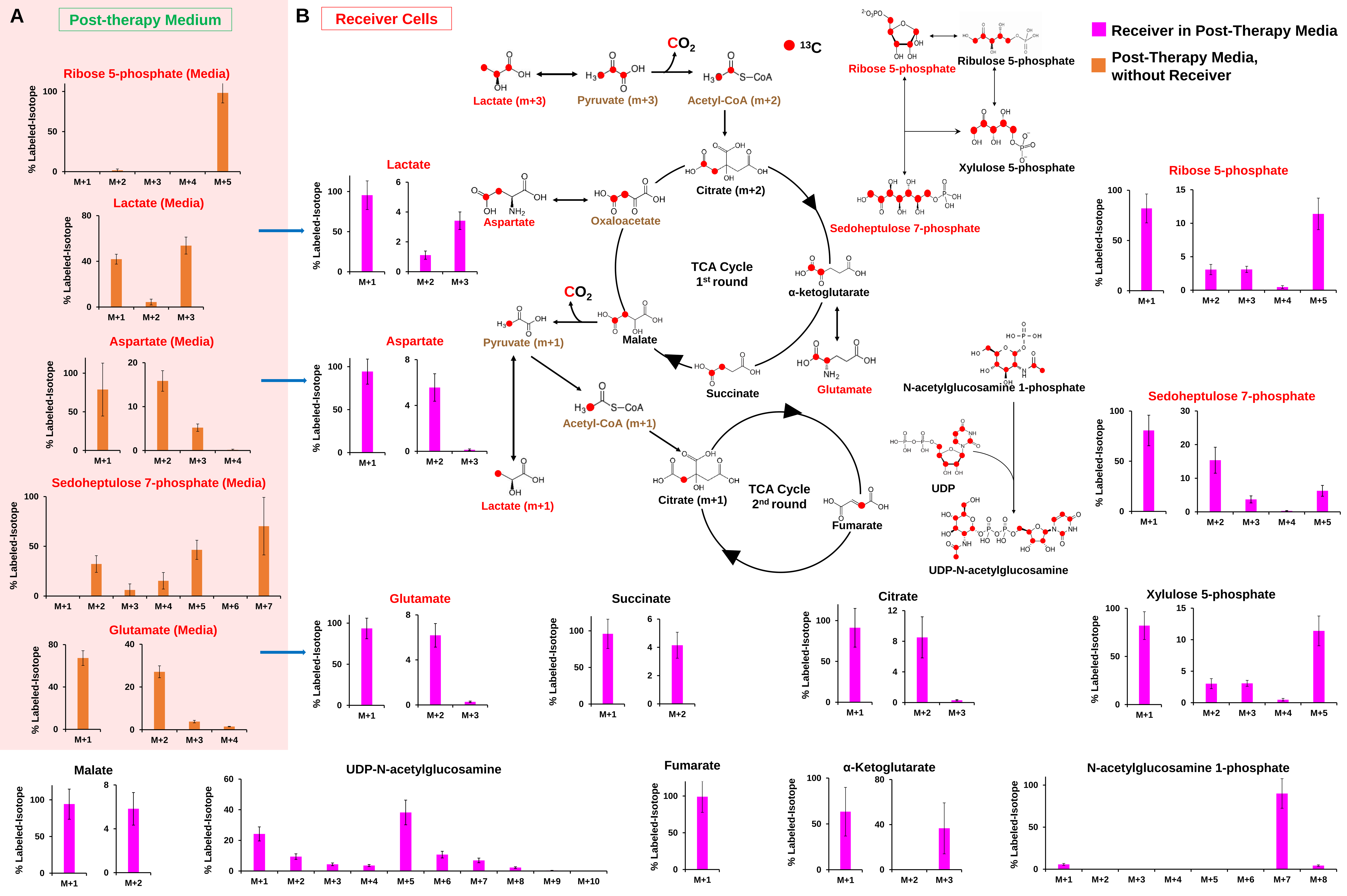

Figure S3. Percentage of ^{13}C-Labeled-Isotopes of Carbohydrate and Hexosamine Pathway Metabolites in (A) Post-Therapy Medium Released from dead Donor Cells after UV Irradiation (B) Receiver Cells Grown in Post-Therapy Medium and Illustration of the Corresponding Pathways. Receiver cells were grown in post-therapy medium containing the dead Donor cell debris (previously grown with ^{13}C-labeled glucose) for 72 hours. Percentages of ^{13}C-labeled-isotope of metabolites found in post-therapy medium containing the dead Donor cell debris (previously grown with ^{13}C-labeled glucose) are shown as orange bars; percentages of ^{13}C-labeled-isotope of Receiver cells grown in post-therapy medium are shown as pink bars. Metabolites found in both the post-therapy medium and the Receiver cells (lactate, aspartate, glutamate, ribose 5-phosphate, and sedoheptulose 7-phosphate) are shown in red. Metabolites found only in the Receiver cells are shown in black. Red dots represent labeled ^{13}C. The % isotope enrichment was calculated by dividing the intensity of each isotopologue by intensities of total labeled isotopologues times 100%. Data are normalized to protein concentration and are shown as mean ± SEM (n = 4 for Receiver cells grown in control medium, n = 5 for Receiver cells grown in post-therapy medium). The experiments were replicated twice with similar results. Data from one set of experiments are shown.

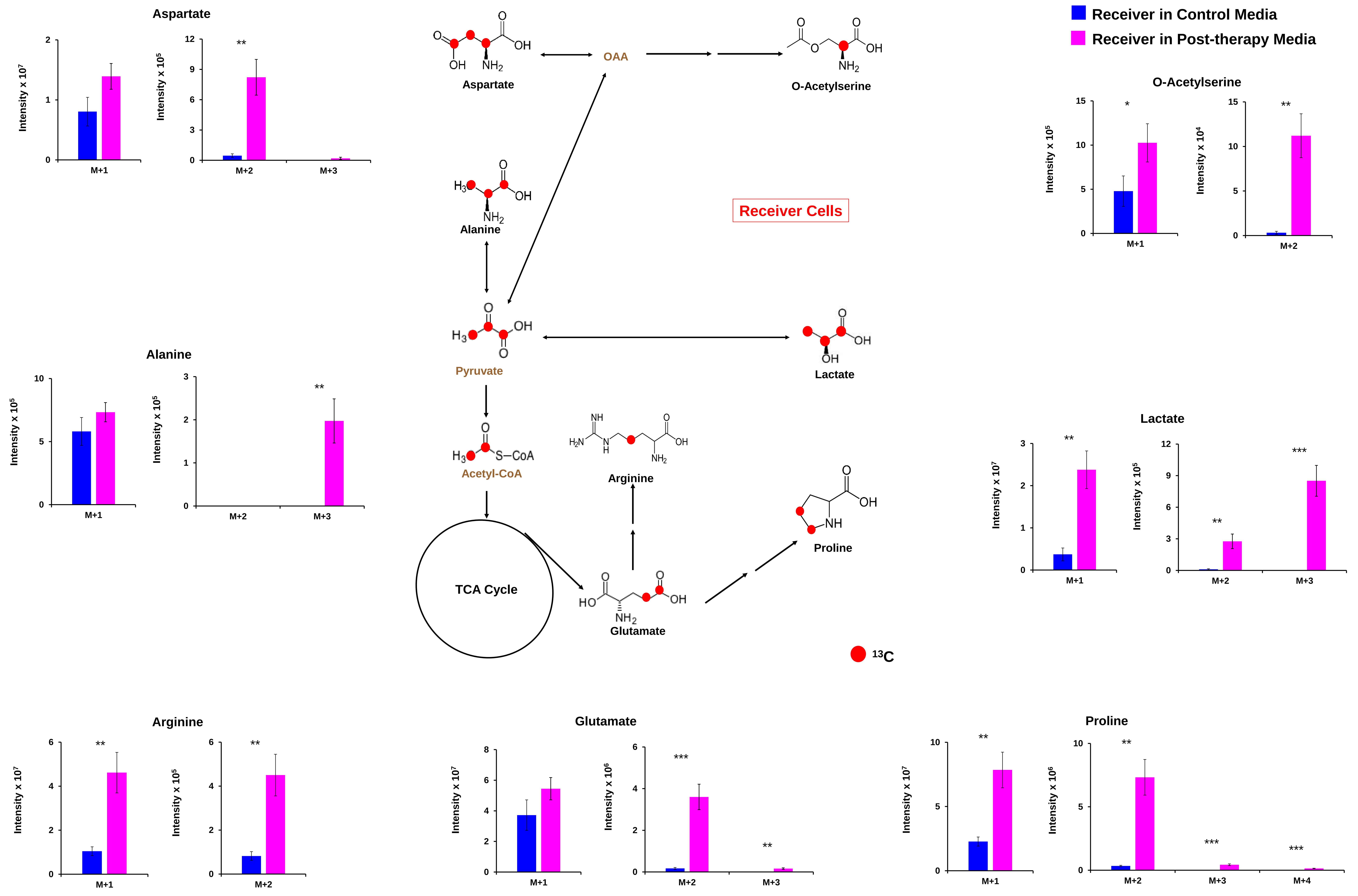

Figure S4. Isotopologues of ^{13}C-Labeled Amino Acid Metabolites of Receiver Cells Grown in Post-Therapy Medium or Control Medium and Illustration of the Corresponding Amino Acid Metabolism Pathways. Metabolite intensities of each isotopologue of Receiver cells grown in post-therapy medium containing the dead Donor cell debris (previously grown with ^{13}C-labeled glucose) for 72 hours are shown as pink bars; metabolite intensities of each isotopologue of Receiver cells grown in control medium are shown as blue bars. Metabolites found in receiver cells are shown in black. Red dots represent labeled ^{13}C. Data are normalized to protein concentration and are shown as mean ± SEM (n = 4 for Receiver cells grown in control medium, n = 5 for Receiver cells grown in post-therapy medium). $^{*}p < 0.05$, $^{**}p < 0.01$, $^{***}p < 0.001$ (Student's *t*-test) where indicated. The experiments were replicated twice with similar results. Data from one set of experiments are shown.

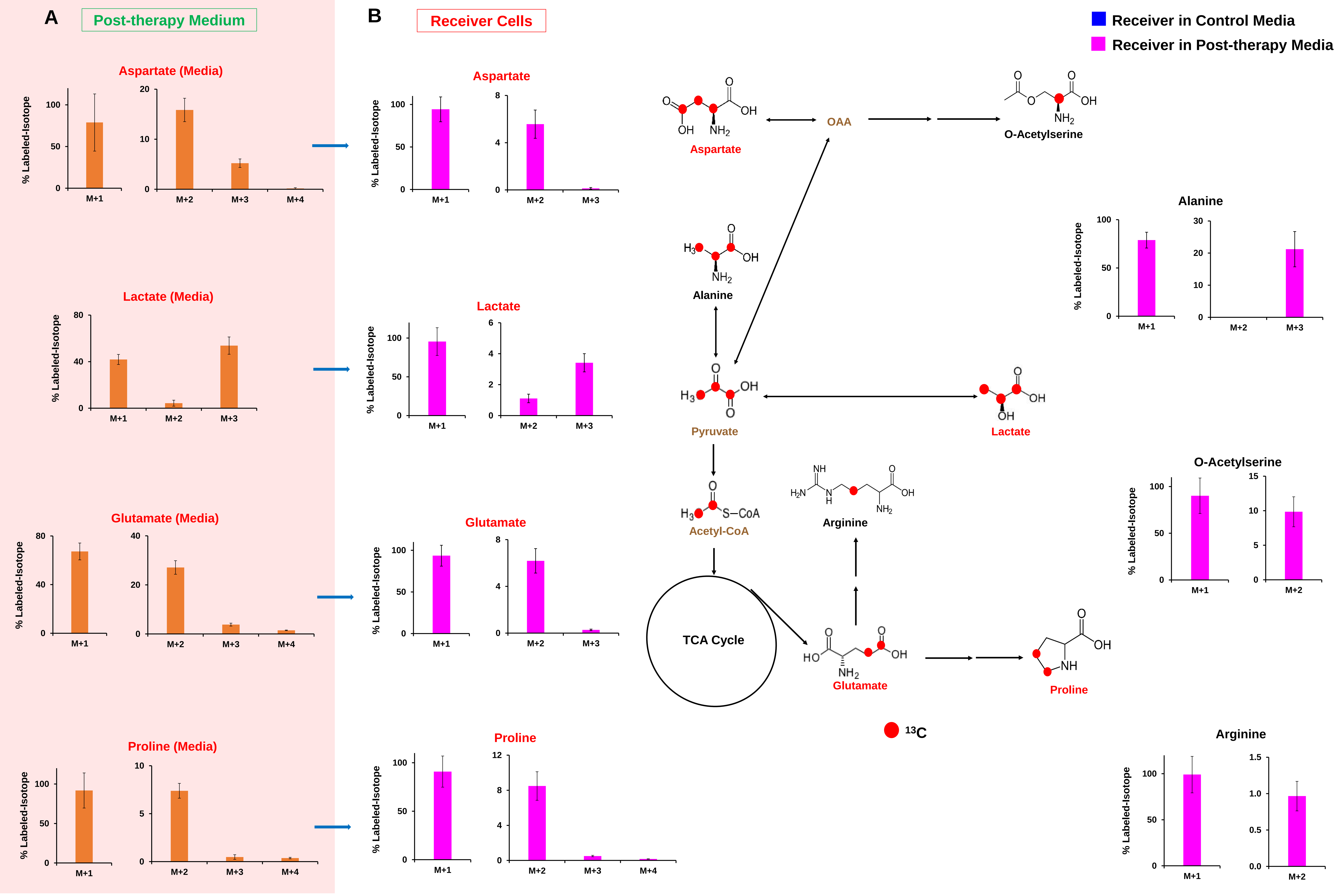

Figure S5. Percentage of ^{13}C-Labeled-Isotopes of Amino Acid Metabolites in (A) Post-Therapy Medium Released from dead Donor Cells after UV Irradiation (B) Receiver Cells Grown in Post-Therapy Medium and Illustration of the Corresponding Amino Acid Metabolism Pathways. Receiver cells were grown in post-therapy medium containing the dead Donor cell debris (previously grown with ^{13}C-labeled glucose) for 72 hours. Percentages of ^{13}C-labeled-isotope of metabolites found in post-therapy medium containing the dead Donor cell debris (previously grown with ^{13}C-labeled glucose) are shown as orange bars; percentages of ^{13}C-labeled-isotope of Receiver cells grown in post-therapy medium are shown as pink bars. Metabolites found in both the post-therapy medium and the Receiver cells (lactate, aspartate, glutamate, and proline) are shown in red. Metabolites found only in the Receiver cells are shown in black. Red dots represent labeled ^{13}C. The % isotope enrichment was calculated by dividing each isotopologue by total labeled isotopologues times 100%. Data are normalized to protein concentration and are shown as mean ± SEM (n = 4 for Receiver cells grown in control medium, n = 5 for Receiver cells grown in post-therapy medium). The experiments were replicated twice with similar results. Data from one set of experiments are shown.

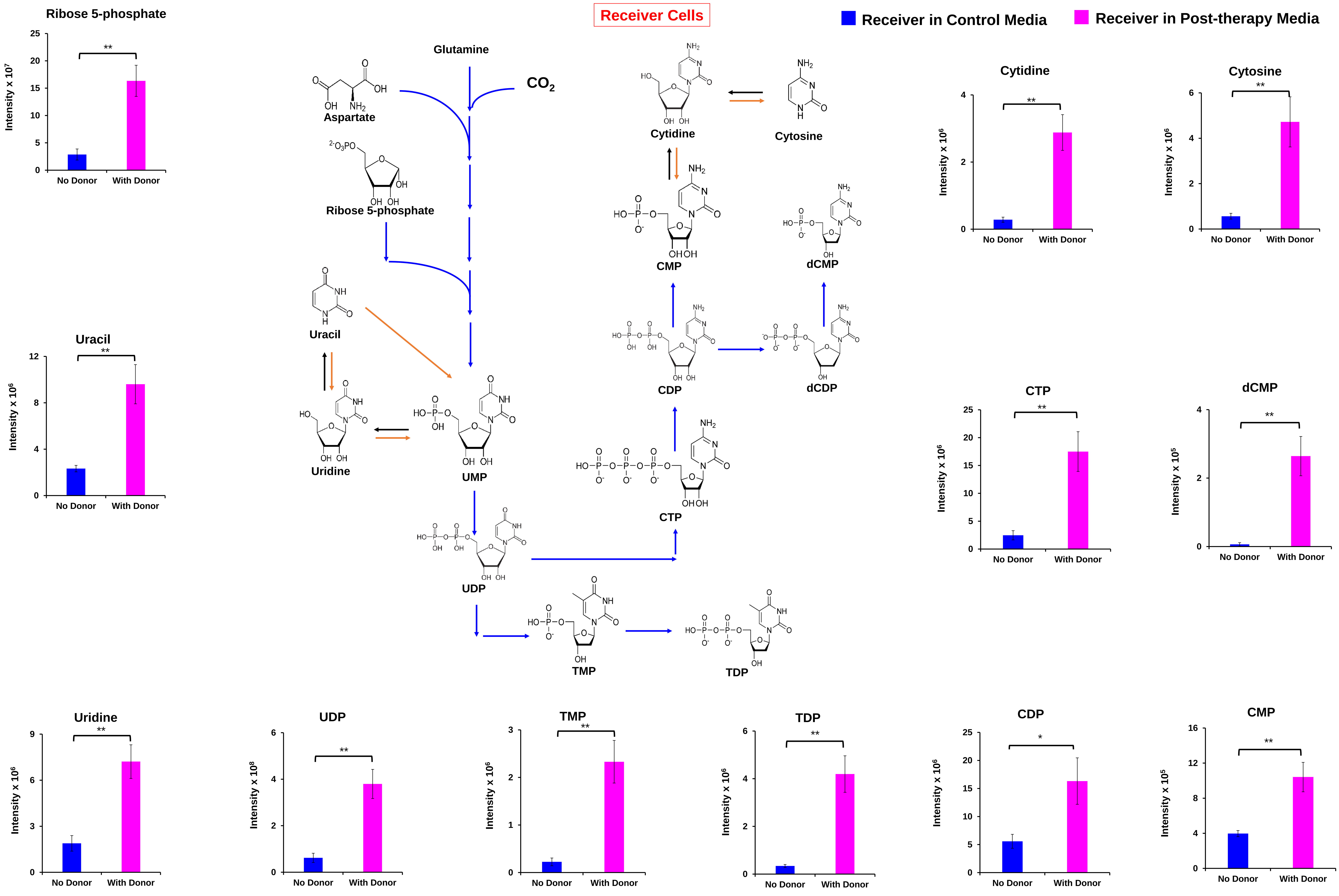

Figure S6. Intensities of Non-Labeled Pyrimidine Metabolites in Receiver Cells Grown in Post-Therapy Medium or Control Medium and Illustration of the Corresponding Pyrimidine Metabolism Pathways. Metabolite intensities of Receiver cells grown in post-therapy medium containing the dead Donor cell debris for 72 hours are shown as pink bars; metabolite intensities of Receiver cells grown in control medium are shown as blue bars. Metabolites are shown in black. Blue arrows indicate *de novo* synthesis, black arrows indicate catabolism, and orange arrows indicate salvage of the pyrimidine metabolites. Data are normalized to protein concentration and are shown as mean ± SEM (n = 4 for Receiver cells grown in control medium, n = 5 for Receiver cells grown in post-therapy medium). *p < 0.05, **p < 0.01, ***p < 0.001 (Student's *t*-test) where indicated. The experiments were replicated twice with similar results. Data from one set of experiments are shown.

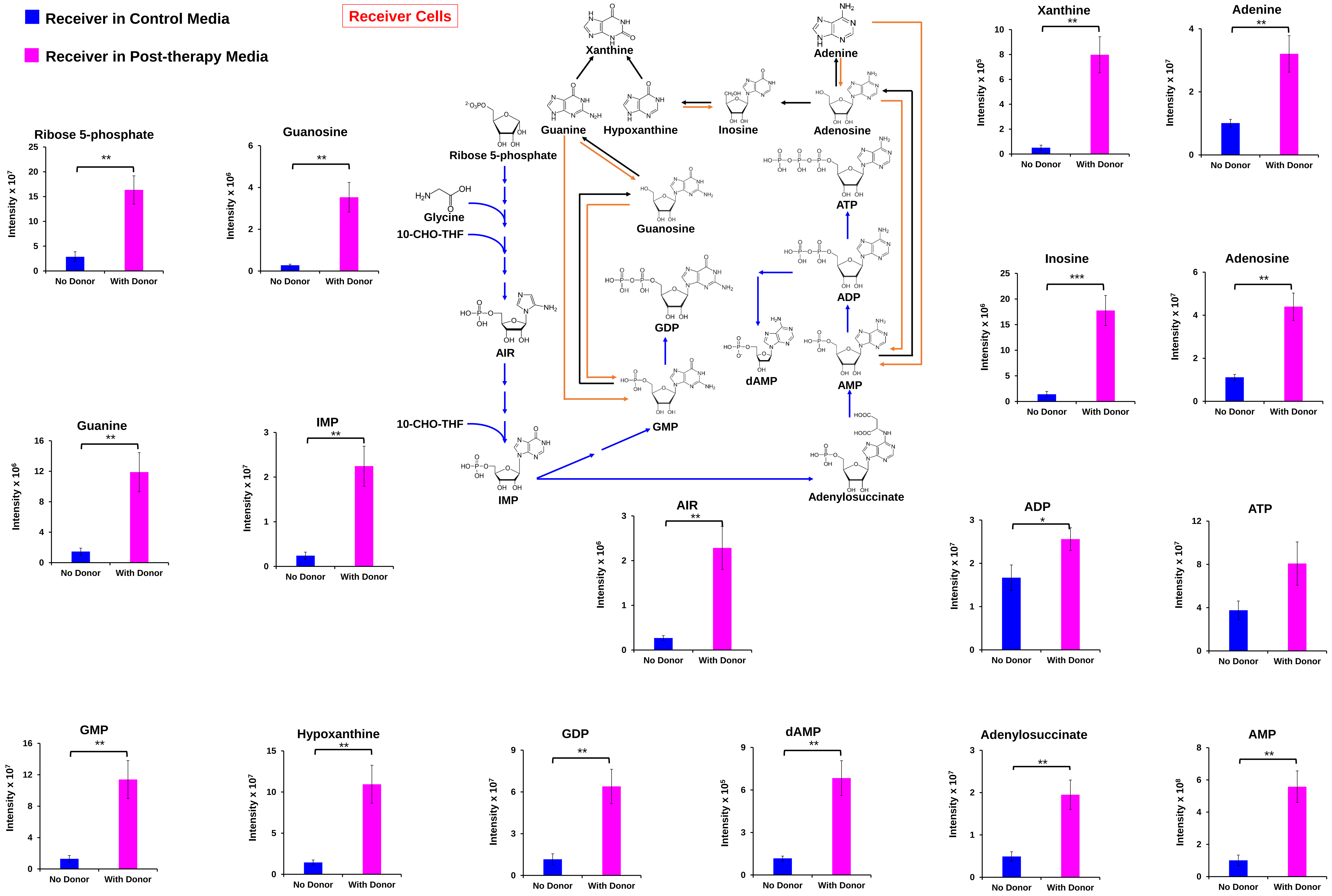

Figure S7. Intensities of Non-Labeled Purine Metabolites in Receiver Cells Grown in Post-Therapy Medium or Control Medium and Illustration of the Corresponding Purine Metabolism Pathways. Metabolite intensities of Receiver cells grown in post-therapy medium containing the dead Donor cell debris for 72 hours are shown as pink bars; metabolite intensities of Receiver cells grown in control medium are shown as blue bars. Metabolites are shown in black. Blue arrows indicate *de novo* synthesis, black arrows indicate catabolism, and orange arrows indicate salvage of the purine metabolites. Data are normalized to protein concentration and are shown as mean ± SEM (n = 4 for Receiver cells grown in control medium, n = 5 for Receiver cells grown in post-therapy medium). $^*p < 0.05$, $^{**}p < 0.01$, $^{***}p < 0.001$ (Student's *t*-test) where indicated. The experiments were replicated twice with similar results. Data from one set of experiments are shown.

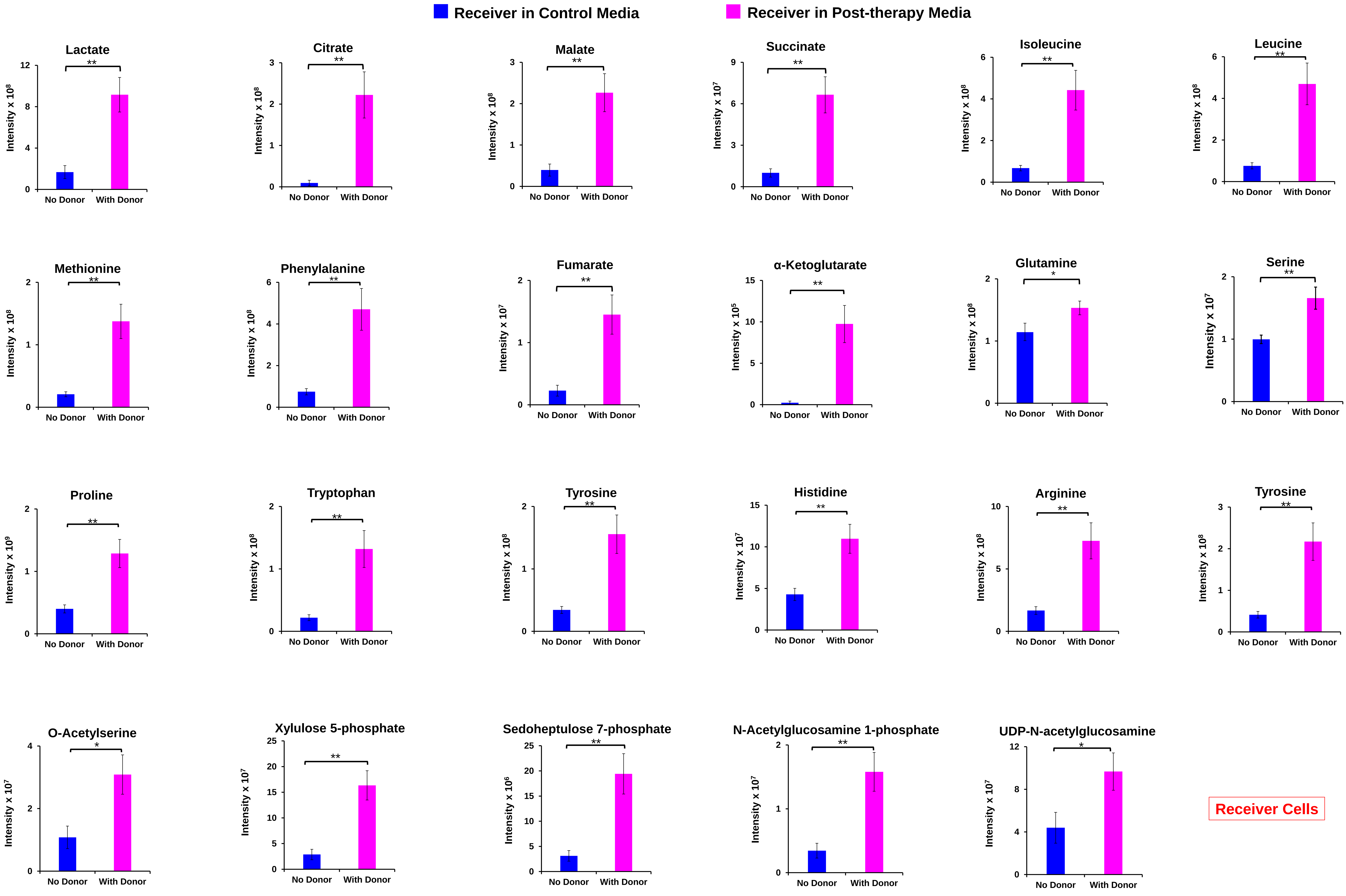

Figure S8. Intensities of Non-Labeled Carbohydrate, Hexosamine Pathway, and Amino Acids Metabolites Receiver Cells Grown in Post-Therapy Medium or in Control Medium. Metabolite intensities of Receiver cells grown in post-therapy medium containing the dead Donor cell debris for 72 hours are shown as pink bars; metabolite intensities of Receiver cells grown in control medium are shown as blue bars. Data are normalized to protein concentration and are shown as mean ± SEM (n = 4 for Receiver cells grown in control medium, n = 5 for Receiver cells grown in post-therapy medium). Only the metabolites which are statistically significant are presented. *$p < 0.05$, **$p < 0.01$, ***$p < 0.001$ (Student's *t*-test) where indicated. The experiments were replicated twice with similar results. Data from one set of experiments are shown.

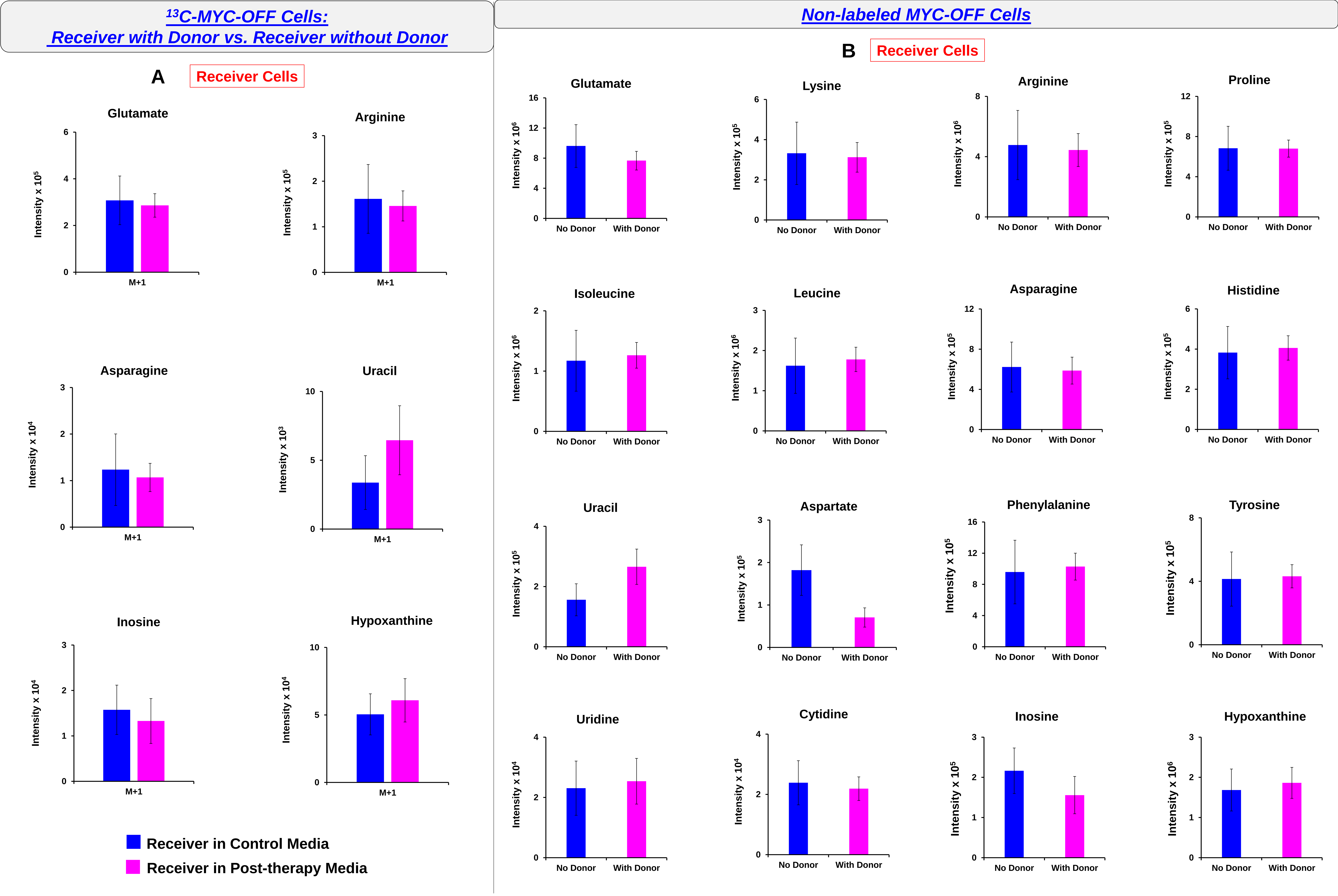

Figure S9. Intensities of Isotopologues of ^{13}C Labeled (A) and Non-Labeled (B) Pyrimidine, Purine, Carbohydrate, and Amino Acid Metabolites in *MYC*-OFF Receiver Cells Grown in Post-Therapy Medium or Control Medium. Metabolite intensities of each isotopologue detected (A) and non-labeled metabolite intensities (B) of Receiver cells grown in post-therapy medium containing the dead Donor cell debris for 72 hours are shown as pink bars; metabolite intensities of each isotopologue detected (A) and non-labeled metabolite intensities (B) of Receiver cells grown in control medium are shown as blue bars. Data are normalized to protein concentration and are shown as mean ± SEM (n = 4 per group).
